# Prognostic value of a non-contrast CT-based radiomics model in patients with Type-B aortic dissection after thoracic endovascular aortic repair

**DOI:** 10.12669/pjms.42.8.16527

**Published:** 2026-08

**Authors:** Jiajun Zou, Xinyi Shi, Jianqi Ni, Qin Jin, Haofeng Ding, Yifeng Shen

**Affiliations:** 1 Jiajun Zou Department of Radiology, The First Hospital of Jiaxing, Affiliated Hospital of Jiaxing University, Jiaxing, Zhejiang Province 314000, P.R. China; 2 Xinyi Shi Department of Radiology, The First Hospital of Jiaxing, Affiliated Hospital of Jiaxing University, Jiaxing, Zhejiang Province 314000, P.R. China; 3 Jianqi Ni Department of Vascular Surgery, The First Hospital of Jiaxing, Affiliated Hospital of Jiaxing University, Jiaxing, Zhejiang Province 314000, P.R. China; 4 Qin Jin Department of Vascular Surgery, The First Hospital of Jiaxing, Affiliated Hospital of Jiaxing University, Jiaxing, Zhejiang Province 314000, P.R. China; 5 Haofeng Ding, Department of Basic Medical, Chengnan Community Healh Service center, Affiliated to Jiaxing Medical University, Jiaxing, Zhejiang Province 314000, P.R. China; 6 Yifeng Shen Department of Vascular Surgery, The First Hospital of Jiaxing, Affiliated Hospital of Jiaxing University, Jiaxing, Zhejiang Province 314000, P.R. China

**Keywords:** B-Type-Aortic dissection, Computer tomography imaging, Nomogram, Poor prognosis, Radiomics, Thoracic endovascular aortic repair

## Abstract

**Objectives:**

To explore the value of a non-contrast computed tomography (CT)-based radiomics model in predicting adverse prognosis in patients with type B aortic dissection (TBAD) after thoracic endovascular aortic repair (TEVAR).

**Methodology:**

A retrospective analysis included data from 107 patients with TBAD undergoing TEVAR at the First Hospital of Jiaxing from January 2010 to May 2024. Patients were randomly divided into a training (n=86) and a validation cohort (n=21) at an 8:2 ratio. Radiomic features were extracted from non-contrast CT images to build a radiomic model. Univariate and multivariate logistic regression analyses identified independent clinical risk factors for constructing a clinical model. Lasso regression was used for data dimensionality reduction and model building. A clinical-radiomic model was established by integrating clinical risk factors with radiomic features. The discriminative ability, calibration, and clinical utility of the three models were evaluated using ROC curves (specificity and sensitivity), calibration curves, and clinical decision curve analysis (DCA), respectively.

**Results:**

Ten radiomic features were selected in the training cohort. Clinical, radiomic, and clinical-radiomic models were generated using eight machine learning algorithms and exhibited good predictive performance. The clinical-radiomic model had the highest AUC values (0.943 in the training cohort, 0.875 in the validation cohort) compared to the clinical and radiomic models. Calibration curves and the Hosmer-Lemeshow test confirmed good calibration of all three models in both sets. DCA revealed that the nomogram model provided favorable clinical net benefits within a certain threshold range.

**Conclusions:**

A non-contrast CT-based radiomics machine learning model has high value for predicting adverse prognosis after TEVAR.

## INTRODUCTION

With the rising incidence of hypertension and atherosclerosis, the prevalence of aortic dissection, a life-threatening acute cardiovascular emergency, has increased markedly in recent years.^1^,^2^ Aortic dissection is caused by various etiologies that lead to an intimal tear in the aortic wall, allowing blood to enter the aortic media.^3^ The resulting separation of the intimal and adventitial layers and propagation of the dissection longitudinally along the aortic axis ultimately forms true and false lumens.^4^ Patients usually present with a sudden, sharp pain, and the mortality rate may reach up to 50% if effective treatment is not administered within 48 hours.^1^,^2^,^4^

Treatment for type B aortic dissection (TBAD) includes open artificial vascular replacement, endovascular therapy, and pharmacotherapy.^5^ Thoracic endovascular aortic repair (TEVAR), a minimally invasive procedure, has now become the first-line treatment for TBAD.^6^ The fundamental principle of TEVAR for TBAD is to cover the primary entry tear with an endovascular stent-graft, occlude the blood supply to the false lumen, reconstruct the true lumen, and ultimately induce thrombosis of the false lumen, thereby achieving vascular remodeling, restoring true lumen perfusion, and stabilizing the main aortic trunk.^7^ Multiple studies have demonstrated that TEVAR may yield better outcomes in aortic remodeling and long-term prognosis for patients with uncomplicated TBAD compared with optimal medical therapy.^8^

However, in some patients with TBAD, TEVAR is associated with poor clinical outcomes and complications, including thoracic/abdominal aortic aneurysmal dilatation.^9^ Adverse events may also occur post-TEVAR, and include complications, such as endoleak, retrograde Stanford Type-A aortic dissection, stent graft-induced new entry (SINE), and neurological ischemia (stroke or paraplegia).^10^ These events can lead to persistent false lumen dilatation, formation of dissecting aortic aneurysm, arterial rupture or organ ischemia, which in turn increases the risks of reoperation and aorta-related mortality, and severely impairs therapeutic efficacy and clinical prognosis. Delayed treatment may even result in aortic rupture and death.^11^,^12^

At present, few studies have examined adverse events following TEVAR for TBAD, and no research has established a non-contrast computed tomography (CT)-based radiomics prediction model using clinical and imaging data from patients with TBAD to assist clinicians in predicting the risk of post-TEVAR adverse events. This study aimed to explore and identify the risk factors for adverse events after TEVAR in patients with TBAD, and construct a predictive model for adverse prognosis by integrating radiomics.

## METHODOLOGY

The study retrospectively enrolled 107 patients with clinically diagnosed TBAD at the First Hospital of Jiaxing from January 2010 to May 2024. All patients underwent non-contrast CT and CT angiography (CTA) prior to any surgical intervention or TEVAR.

### Ethical approval:

The study was approved by Ethics Committee/IRB of the institution (No. 2023-KY-406-002; Dated: July 11, 2025). Informed consent was waived because of the retrospective nature of the study and the clinical data was anonymized.

### Inclusion criteria:

- Completion of an emergency non-contrast CT scan within two weeks after onset, with complete clinical data available.
- No major adverse events during the acute phase.
- No repeat aortic surgery or TEVAR performed before the follow-up aortic CTA examination.
- CT findings indicate no history of major vascular intervention or surgical treatment prior to the procedure.
- Underwent and completed TEVAR surgery.

### Exclusion criteria:

- Age < 18 years old or > 90 years old.
- Chronic aortic dissection.
- Poor image quality and large analysis errors.
- Pregnant women.
- Marfan syndrome or other connective tissue diseases.
- Syphilis or other inflammatory diseases of the aorta.
- Patients with death, rupture, or re-intervention before completion of baseline imaging evaluation.

### Treatment:

All participants received TEVAR combined with effective antihypertensive medication. TEVAR was performed in accordance with standard procedural protocols. Technical success was defined as successful deployment of the stent graft at the intended target location in the absence of a Type-I or Type-III endoleak, open surgical conversion, death within 24 hours, or stent obstruction or migration; and intraoperative angiography confirmed complete isolation of the lesion.^13^,^14^ Blood pressure was aggressively controlled, with systolic blood pressure maintained between 100 and 120 mmHg during the perioperative period. At discharge, all patients were educated by clinicians regarding the potential risks of aortic dissection, daily living precautions, and the necessity of regular follow-up visits.

### Follow-up:

To ensure the quality of follow-up data, patients were scheduled for follow-up at one month, three months, and 12 months after TEVAR, followed by annual follow-up thereafter, with the follow-up cut-off date set as November 2025. All patients were advised to return to our hospital for reexamination at the scheduled intervals. Regular telephone follow-up was conducted to minimize data loss and patient dropout. For patients who missed scheduled visits, telephone contact was attempted with the patients themselves, their designated relatives or contacts, or their attending physicians. If patients were unable to visit the First Hospital of Jiaxing, they were advised to seek medical attention at the nearest tertiary hospital, and their reexamination results were tracked promptly. To ensure the authenticity and accuracy of follow-up, patients who did not respond to repeated follow-up attempts were considered lost to follow-up. The follow-up was terminated in the event of adverse events after TEVAR, including endoleak, retrograde Stanford Type-A aortic dissection, stent-graft-induced new entry, and neurological ischemia (stroke or paraplegia).

### Selection and Clinical Characteristics and Coding:

To construct a prognostic prediction model for TBAD and optimize its completeness and efficiency, the following clinical characteristics were incorporated: gender, age, body weight, history of hypertension, C-reactive protein, history of diabetes mellitus, smoking history, D-dimer, fibrinogen, low-density lipoprotein, cholesterol, and aortic diameter. Given the large volume of data and the complex parameters required for this study, all parameters were coded to improve computational efficiency and the visual clarity of presentation. All categorical variables were binary encoded for model construction.

### CT Image scanning:

All patients underwent CT scanning using a Canon 320-row CT scanner. The scanning parameters were set at 100–120 kVp and automatic mAs. The scanning range for non-contrast and contrast-enhanced CT covered from the thoracic inlet to the pubic symphysis. Images were uploaded to the Picture Archiving and Communication System (PACS) in DICOM format, and all patient image data were downloaded from the PACS. In cases of disagreement, images were reviewed together by both physicians to adjudicate the results.

### Delineation of Regions of Interest (ROI):

Non-contrast CT images were imported into ITK-SNAP software (Version 3.8.0, www.itksnap.org). After adjusting the window width and window level to optimal settings, a radiologist with more than three years of CT diagnostic experience, who was blinded to the patients’ clinical outcomes, manually delineated the ROI along each axial slice of the aorta. All slice-level ROIs were ultimately fused to generate a 3D contour. The delineation results were subsequently reviewed and verified by another physician with over three years of clinical experience in vascular surgery.

### Extraction of Radiomic features:

To eliminate the impact of inter-image variability, image resampling was first performed. Radiomic features were extracted from the images using PyRadiomics (a Python-based library, version 3.0.1). The extracted feature parameters included: first-order features, shape features (shape 2D, shape 3D), gray level run-length matrix (GLRLM) features, gray level dependence matrix (GLDM) features, gray level size zone matrix (GLSZM) features, neighboring gray tone difference matrix (NGDTM) features, gray level co-occurrence matrix (GLCM) features, and filtered features.

### Selection of Radiomic features:

First, invalid feature data were removed from the dataset. Missing values were imputed using mean imputation, and data were standardized using the Z-score method. Feature selection and model construction were performed exclusively on the training cohort, while the test set was used only for internal model validation. The feature dataset was randomly divided into a training cohort and a validation cohort in an 8:2 ratio using a random selection function. Next, feature screening was performed using the t-test or Mann-Whitney U test (U-test). Subsequently, the Pearson correlation coefficient (PCC) was calculated for each pair of features; if the PCC was ≥ 0.9, the feature with the lower P-value for intergroup differences was retained. Least absolute shrinkage and selection operator (Lasso) regression, combined with 10-fold cross-validation, was applied to perform dimensionality reduction on the radiomic feature data from the training cohort. Radiomic features with non-zero coefficients were extracted to form the final feature set.

### Construction and validation of predictive models:

The training cohort was separately input into eight machine learning algorithms, namely Logistic Regression (LR), Support Vector Machine (SVM), K-Nearest Neighbor (KNN), Random Forest, Extra Trees, XGBoost, LightGBM and Multi-Layer Perceptron (MLP), to develop the corresponding radiomic model, clinical model, and combined model (radiomic features + clinical characteristics), with the validation cohort used for internal verification. Receiver operating characteristic (ROC) curves were plotted to evaluate the diagnostic performance of each model. The area under the ROC curve (AUC) with its 95% confidence interval (CI), sensitivity, specificity, positive predictive value (PPV), negative predictive value (NPV), accuracy, and F1-score were calculated for both the training and validation cohorts. A nomogram model was constructed, and calibration curves and decision curve analysis (DCA) were further used for model evaluation. The discrepancy between the actual and predicted probabilities was further assessed using the Hosmer-Lemeshow test. DCA was performed using Python. To further compare the inter-model differences at a deeper level, the Delong test was used to evaluate the performance of the respective ROC curves.

### Statistical analysis:

All statistical analyses were performed using IBM SPSS Statistics 23.0 software. Categorical variables were reported as the number of cases and percentages, and compared using the Chi-square test or Fisher’s exact test. For quantitative variables, those with a normal distribution were reported as mean ± standard deviation and analyzed using Student’s t-test; non-normally distributed data were reported as median (interquartile range, IQR) and analyzed using the Mann-Whitney U test. A two-tailed P-value < 0.05 was considered statistically significant.

## RESULTS

In the training cohort of 86 patients who underwent TEVAR, 20 were classified as adverse-prognosis and 66 as non-adverse-prognosis. In the validation cohort of 21 TEVAR patients, five were classified as adverse-prognosis and 16 as non-adverse-prognosis. As shown in Table-I, the adverse prognosis group of the training cohort had a significantly larger aortic diameter (46.10±15.34 mm) than the non-adverse prognosis group (28.32±9.37 mm), a higher mean age (66.80±7.70 years vs. 54.58±12.48 years), and a higher D-dimer level (7940 [3390, 9320] ng/mL vs. 2312 [1090, 4930] ng/mL) (P<0.05). No statistically significant differences were observed in other clinical data and conventional CT features between the two groups (P>0.05).

**Table-I:** Clinical characteristics of the adverse-prognosis group and the non-adverse-prognosis group (training cohort).

Index	Non-adverse prognosis (n=66)	Adverse prognosis (n=20)	P
***Aortic diameter, mean ± SD***	28.32±9.37	46.10±15.34	<0.001
Age, mean ± SD	54.58±12.48	66.80±7.70	<0.001
D-dimer, median(IQR)	2312[1090, 4930]	7940[3390, 9320]	0.028
Fibrinogen, mean ± SD	4.80±1.80	4.20±3.03	0.187
Low density lipoprotein, mean ± SD	2.54±0.84	2.63±0.63	0.674
Cholesterol, mean ± SD	4.21±0.96	4.13±0.78	0.719
Crp, median(IQR)	6.90[3.00, 27.20]	6.40[5.00, 48.90]	0.747
***Calcification ingression, n (%)***			1
No	33(50.00)	10(50.00)	
Yes	33(50.00)	10(50.00)	
***Internal displacement of intimal film, n (%)***			1
No	18(27.27)	6(30.00)	
Yes	48(72.73)	14(70.00)	
***High attenuation area, n (%)***			0.624
No	47(71.21)	16(80.00)	
Yes	19(28.79)	4(20.00)	
***Pericardial effusion blood, n (%)***			1
No	59(89.39)	18(90.00)	
Yes	7(10.61)	2(10.00)	
***Pleural and abdominal effusion blood, n (%)***			0.344
No	47(71.21)	17(85.00)	
Yes	19(28.79)	3(15.00)	
***Calcification of ascending aorta, n (%)***			0.150
No	62(93.94)	16(80.00)	
Yes	4(6.06)	4(20.00)	
***Calcification of aortic arch, n (%)***			0.799
No	34(51.52)	9(45.00)	
Yes	32(48.48)	11(55.00)	
***Calcification of descending aorta, n (%)***			0.903
No	20(30.30)	7(35.00)	
Yes	46(69.70)	13(65.00)	
***Sex, n (%)***			1
Male	14(21.21)	4(20.00)	
Female	52(78.79)	16(80.00)	
***Hypertension, n (%)***			0.538
No	20(30.30)	4(20.00)	
Yes	46(69.70)	16(80.00)	
***Diabetes, n (%)***			0.370
No	60(90.91)	20(100.00)	
Yes	6(9.09)	0(0.00)	
***Smoking, n (%)***			1
No	52(78.79)	16(80.00)	
Yes	14(21.21)	4(20.00)	

In the validation cohort, the adverse prognosis group had a higher mean age (71.20±8.67 years vs. 55.62±12.44 years), a higher D-dimer level (10780 [7370, 14010] ng/mL vs. 3800 [2080, 4750] ng/mL), and a lower fibrinogen level (2.14±1.15 vs. 4.99±1.48 g/L), with statistically significant differences (P<0.05; Table-II).

**Table-II:** Clinical characteristics of the adverse-prognosis group and the non-adverse-prognosis group (validation cohort).

Index	Non-adverse prognosis (n=16)	Adverse prognosis (n=5)	P value
Aortic diameter, mean ± SD	26.38±7.44	42.60±18.80	0.105
Age, mean ± SD	55.62±12.44	71.20±8.67	0.018
D-dimer, median(IQR)	3800[2080, 4750]	10780[7370, 14010]	0.023
Fibrinogen, mean ± SD	4.99±1.48	2.14±1.15	0.004
Low density lipoprotein, mean ± SD	2.75±0.78	2.96±0.63	0.650
Cholesterol, mean ± SD	4.49±1.11	4.37±0.55	0.807
CRP, median(IQR)	28.10[4.70, 47.20]	5.90[2.60, 23.60]	0.342
***Calcification ingression, n (%)***			1
No	9(56.25)	3(60.00)	
Yes	7(43.75)	2(40.00)	
***Internal displacement of intimal film, n (%)***			0.549
No	3(18.75)	0(0.00)	
Yes	13(81.25)	5(100.00)	
***High attenuation area, n (%)***			0.429
No	15(93.75)	4(80.00)	
Yes	1(6.25)	1(20.00)	
***Pericardial effusion blood, n (%)***			0.238
No	16(100.00)	4(80.00)	
Yes	0(0.00)	1(20.00)	
***Pleural and abdominal effusion blood, n (%)***			1
No	12(75.00)	4(80.00)	
Yes	4(25.00)	1(20.00)	
***Calcification of ascending aorta, n (%)***			
No	16(100.00)	3(60.00)	0.048
Yes	0(0.00)	2(40.00)	
***Calcification of aortic arch, n (%)***			0.598
No	12(75.00)	3(60.00)	
Yes	4(25.00)	2(40.00)	
***Calcification of descending aorta, n (%)***			0.598
No	4(25.00)	2(40.00)	
Yes	12(75.00)	3(60.00)	
***Sex, n (%)***			1
Male	5(31.25)	2(40.00)	
Female	11(68.75)	3(60.00)	
***Hypertension, n (%)***			0.553
No	3(18.75)	2(40.00)	
Yes	13(81.25)	3(60.00)	
***Diabetes, n (%)***			1
No	15(93.75)	5(100.00)	
Yes	1(6.25)	0(0.00)	
***Smoking, n (%)***			0.549
No	13(81.25)	5(100.00)	
Yes	3(18.75)	0(0.00)	

***Note:*** Categorical variables with small expected cell counts were compared using the two-sided Fisher’s exact test.

**Fig.1:**
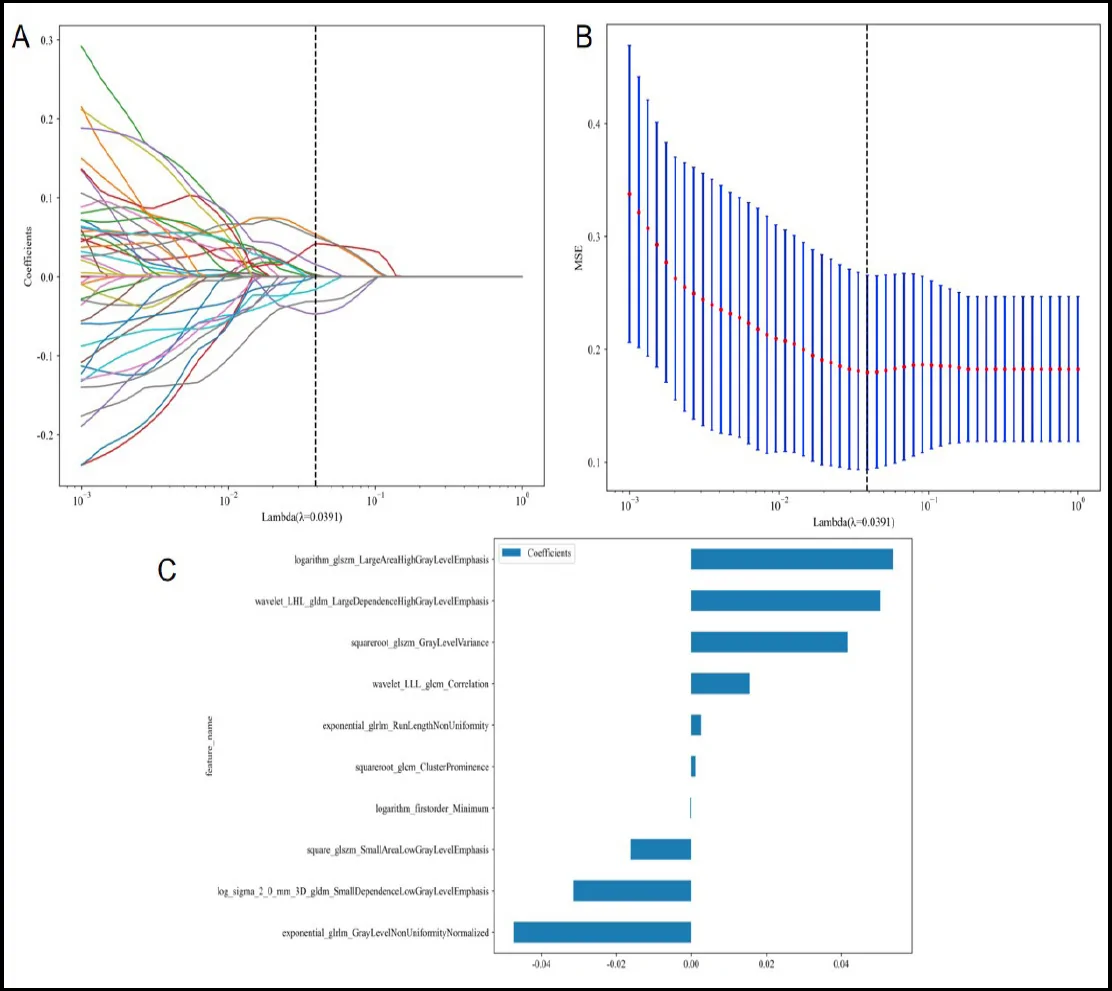
Workflow of radiomic analysis. (A) Curves in different colors represent the change trajectories of each independent variable coefficient in the plot. The ordinate denotes the coefficient value, and the upper abscissa indicates the number of non-zero coefficients in the model at the corresponding time. (B) Ten radiomic features were extracted via the LASSO regression algorithm. (C) The bar graph of 10 radiomics features that achieved nonzero coefficients.

A total of 1561 radiomic features were extracted using PyRadiomics. First, t-tests and Mann-Whitney U tests, as well as correlation coefficient analyses, were performed for initial screening. Lasso regression analysis was subsequently conducted, with 10-fold cross-validation applied to identify the optimal lambda value of 0.0391. Ultimately, 10 radiomic feature parameters with non-zero coefficients were obtained (Fig.1).

**Fig.2:**
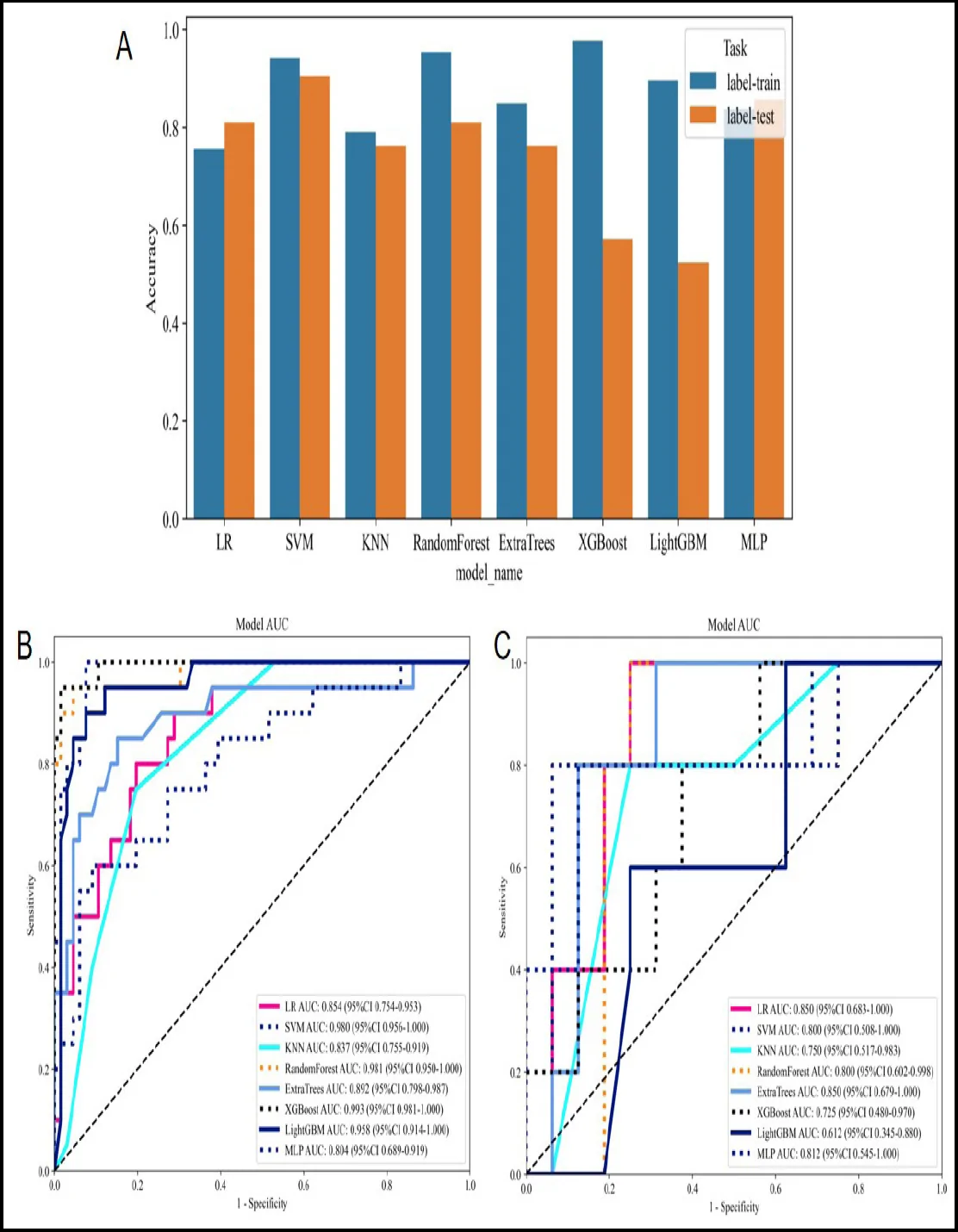
ROC curves of 8 machine learning-based radiomics models. (A) Eight machine learning algorithms. (B) ROCs of the training model. (C) ROCs of the validation model.

**Fig.3:**
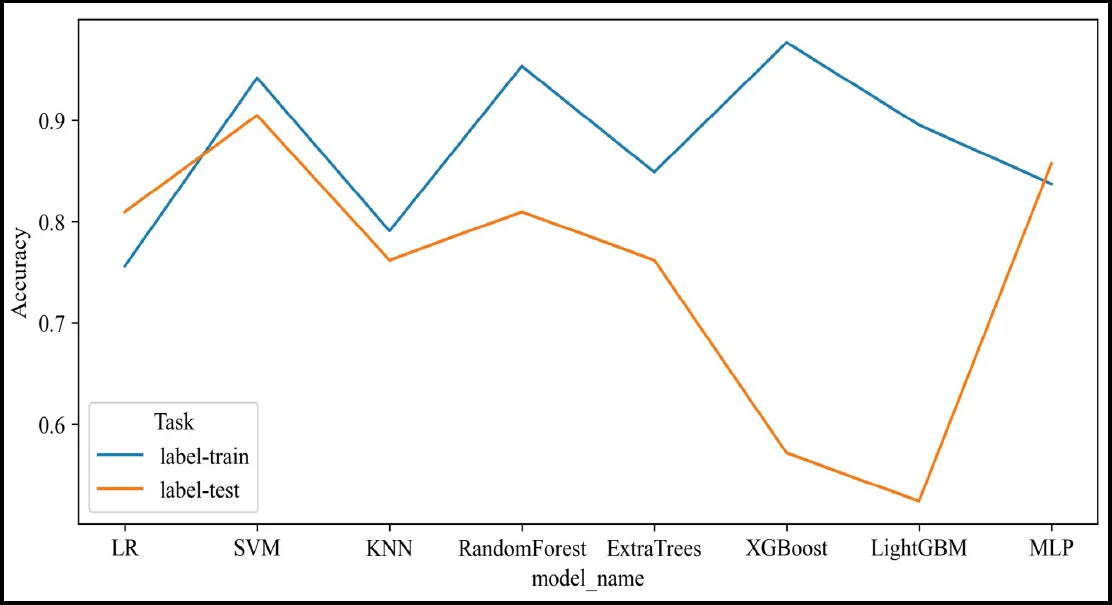
Comparison of the accuracy of eight machine learning models in the training and validation cohorts.

The training cohort was separately input into eight machine learning algorithms, namely LR, SVM, KNN, Random Forest, Extra Trees, XGBoost, LightGBM, and MLP, to develop the corresponding radiomic model, clinical model, and combined model (radiomic features + clinical characteristics), with the validation cohort used for internal verification. The radiomic models constructed by the eight machine learning algorithms all exhibited favorable predictive performance in both cohorts and demonstrated a certain degree of predictive accuracy for evaluating adverse prognosis after TEVAR (Table-III). To intuitively demonstrate the advantages of the models, ROC curves for the validation cohort were plotted in Python (Fig.2). The AUC values of the radiomic models built by the eight machine learning algorithms in the training cohort all exceeded 0.8, indicating that the constructed models had high diagnostic efficacy. A line plot was used to compare the accuracy of the eight machine learning models in the training and validation cohorts (Fig.3).

**Table-III:** Comparison of models constructed by machine learning on the training and the validation cohorts.

Model	Accuracy	AUC	95% CI	Sensitivity	Specificity	PPV	NPV	F1	Task
LR	0.756	0.854	0.7542 - 0.9534	0.9	0.712	0.486	0.959	0.632	train
LR	0.81	0.85	0.6832 - 1.0000	1	0.75	0.556	1	0.714	test
SVM	0.942	0.98	0.9562 - 1.0000	1	0.924	0.8	1	0.889	train
SVM	0.905	0.8	0.5077 - 1.0000	0.8	0.937	0.8	0.937	0.8	test
KNN	0.791	0.837	0.7546 - 0.9189	0.75	0.803	0.536	0.914	0.625	train
KNN	0.762	0.75	0.5171 - 0.9829	0.8	0.75	0.5	0.923	0.615	test
RandomForest	0.953	0.981	0.9502 - 1.0000	0.95	0.955	0.864	0.984	0.905	train
RandomForest	0.81	0.8	0.6025 - 0.9975	1	0.75	0.556	1	0.714	test
ExtraTrees	0.849	0.892	0.7982 - 0.9866	0.85	0.848	0.63	0.949	0.723	train
ExtraTrees	0.762	0.85	0.6788 - 1.0000	1	0.687	0.5	1	0.667	test
XGBoost	0.977	0.993	0.9814 - 1.0000	0.95	0.985	0.95	0.985	0.95	train
XGBoost	0.571	0.725	0.4805 - 0.9695	1	0.437	0.357	1	0.526	test
LightGBM	0.895	0.958	0.9139 - 1.0000	0.95	0.879	0.704	0.983	0.809	train
LightGBM	0.524	0.613	0.3453 - 0.8797	1	0.375	0.333	1	0.5	test
MLP	0.837	0.804	0.6887 - 0.9188	0.6	0.909	0.667	0.882	0.632	train
MLP	0.857	0.812	0.5455 - 1.0000	0.8	0.875	0.667	0.933	0.727	test

**Fig.4:**
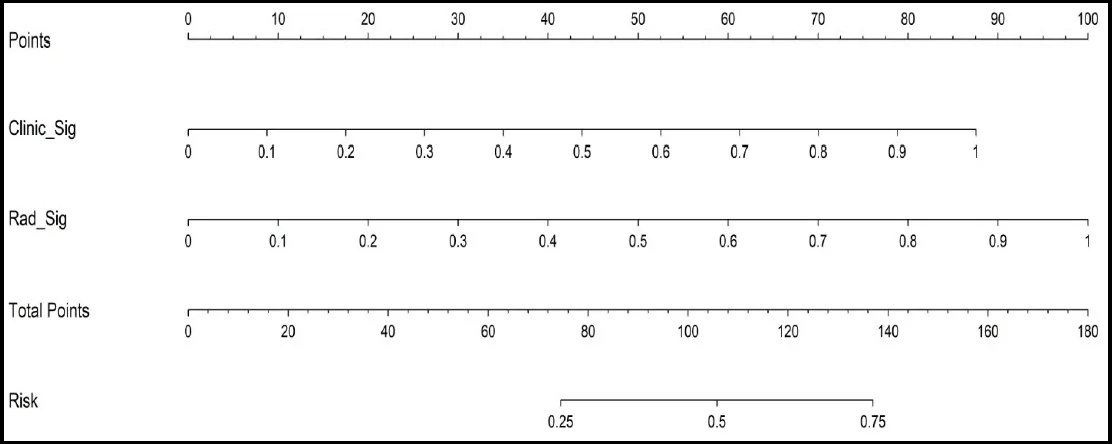
Nomogram model for predicting adverse prognosis after TEVAR. Each level of the predictive variable is assigned a specific score. The total score is generated by summing the scores of all predictive variables, which corresponds to the probability of adverse prognosis.

To better translate the radiomic model into clinical practice and more intuitively demonstrate its advantages, the clinical model was fully integrated with the radiomic model, a clinical-radiomic model was constructed via Logistic regression, and ultimately, its nomogram was plotted (Fig.4).

**Fig.5:**
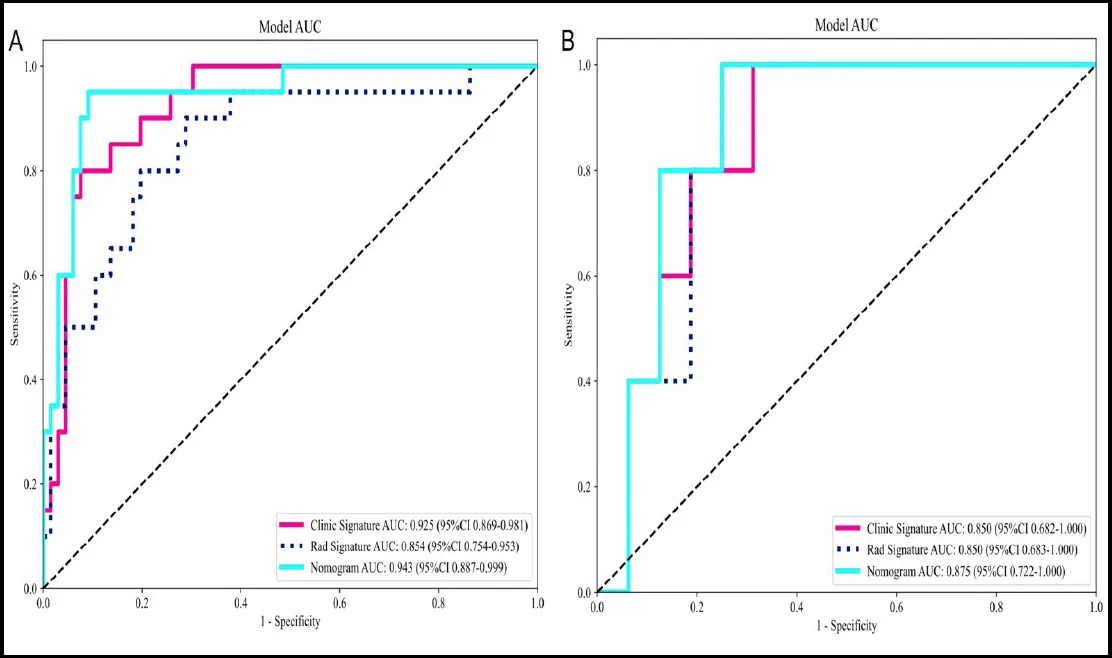
(A) ROC curves of the clinical model, radiomic model, and Nomogram model in the training cohort; (B) ROC curves of the clinical model, radiomic model, and Nomogram model in the validation cohort.

In the training cohort, the nomogram model achieved an AUC of 0.943, the radiomic model an AUC of 0.854, and the clinical model an AUC of 0.925, indicating superior predictive performance for the nomogram model. In the validation cohort, the nomogram model had an AUC of 0.875, the radiomic model of 0.850, and the clinical model of 0.850; the Nomogram model also exhibited better predictive performance (Fig.5).

**Fig.6:**
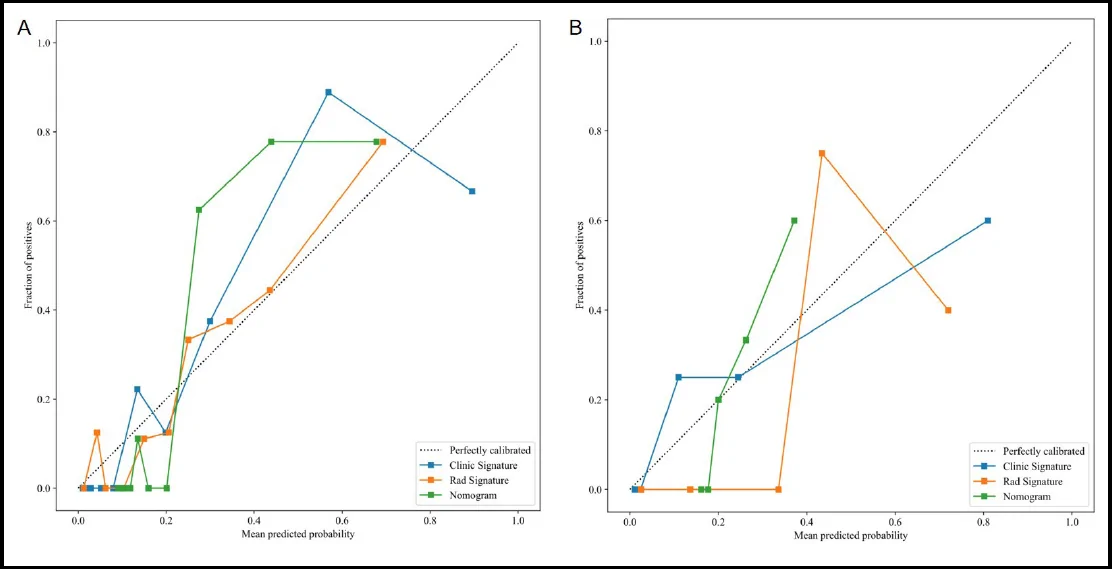
(A) Calibration curve for the training cohort; (B) Calibration curve for the validation cohort. The x-axis represents the predicted probability of adverse prognosis after TEVAR, and the y-axis denotes the observed adverse prognosis. The diagonal dashed line represents perfect calibration, while the three model-specific curves represent the calibration performance of the clinical, radiomic, and nomogram models. A closer fit of the solid line to the diagonal dashed line indicates a more accurate prediction. Greater proximity of a model-specific curve to the diagonal line indicates better calibration.

**Fig.7:**
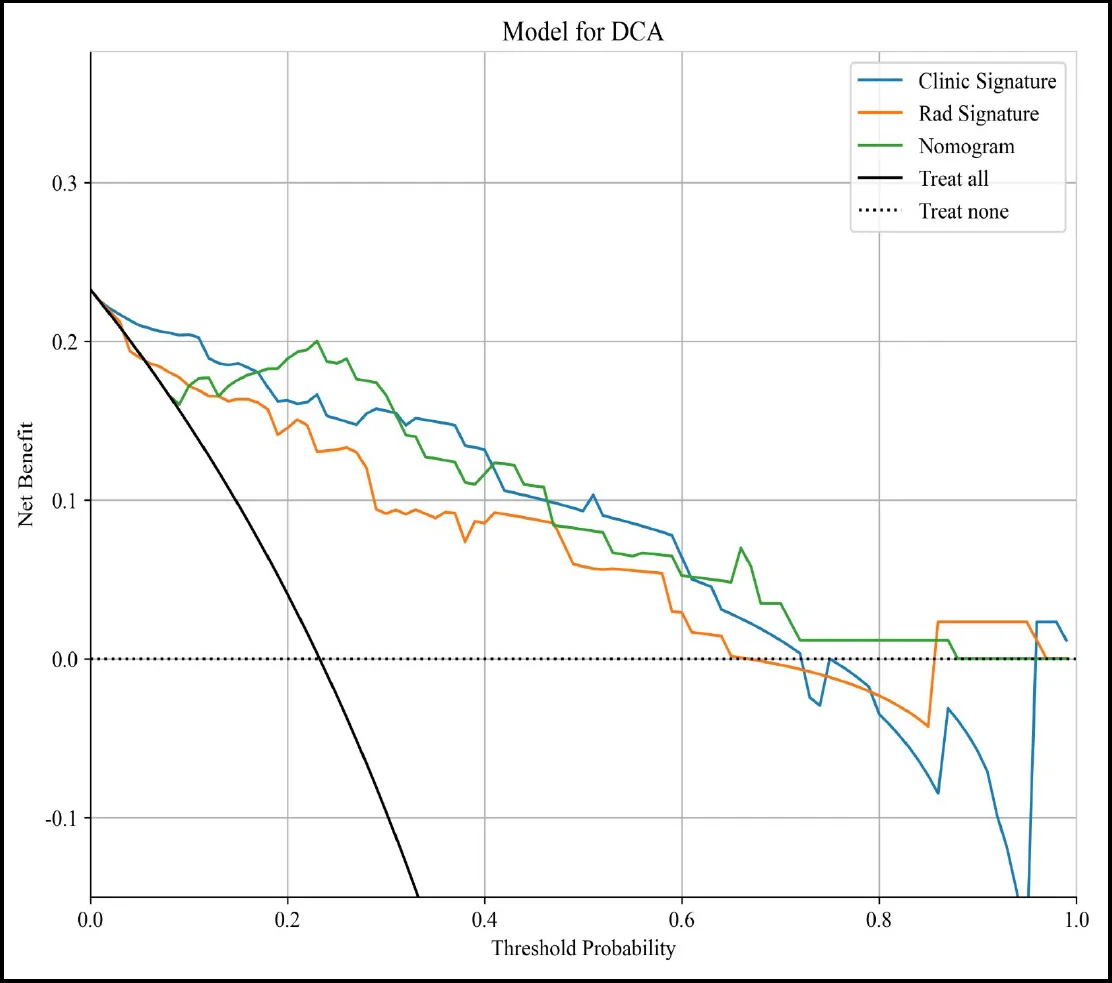
Decision curve analysis of the clinical, radiomic, and nomogram models. The x-axis represents the threshold probability, and the y-axis represents the net benefit. The oblique reference line represents the treat-all strategy, whereas the horizontal line at zero represents the treat-none strategy. A model provides clinical utility when its net-benefit curve lies above both reference strategies within a clinically relevant threshold-probability range.

The calibration curves for the radiomic model, clinical model, and nomogram model were then plotted (Fig.6). Calibration curves reflect the models’ fitting performance: the 45° dashed line represents an ideal predictive model, while the other three solid lines denote each model’s predictive performance, with a closer fit to the dashed line indicating higher model accuracy. The Hosmer-Lemeshow test showed that in both the training and validation cohorts, there were no significant differences between the actual and predicted probabilities of the radiomic model, clinical model, and nomogram model (P>0.05) (Table-IV), indicating good calibration of the models. To verify the clinical application value of the nomogram model, it was compared with the clinical model and the machine-learning-based radiomic model. The DCA curve of the Nomogram model demonstrated its clinical utility (Fig.7).

**Table-IV:** Hosmer-Lemeshow test.

Group	Clinical model	Radiomics model	Nomogram
Training cohort	0.295	0.520	0.124
Validation cohort	0.123	0.396	0.371

The Delong test showed that in the training cohort, the nomogram model exhibited optimal predictive performance and demonstrated a significant advantage over the radiomic model, with a statistically significant difference (P < 0.05). In the validation cohort, no statistically significant differences were observed between the nomogram model and the other models (P > 0.05) (Table-V).

**Table-V:** Delong test.

Group	Nomogram Vs Clinical model	Nomogram Vs Radiomics model	Radiomics model Vs Clinical model
Training cohort	0.377	0.015	0.015
Validation cohort	0.593	0.727	1.000

## DISCUSSION

In this study, three models for predicting adverse prognosis after TEVAR in patients with TBAD were constructed and compared. The nomogram model yielded the highest AUC values in both the training and the validation cohort, demonstrating superior predictive performance relative to the clinical and radiomic models. To more intuitively illustrate the advantages of the Nomogram model, calibration curves of the three models were plotted. The nomogram, radiomic and clinical models all showed good calibration in both cohorts. DCA validation revealed that the nomogram model achieved a favorable clinical net benefit within a certain threshold range.

The Delong test was further applied to compare the AUCs. Delong’s test suggests that the nomogram has a significant advantage over radiomics models in the training cohort (P<0.05), but there is no significant difference compared to other models. There was no statistical difference between the nomogram and other models in the validation cohort. Therefore, future research should expand the predictive performance of the nomogram model by enriching clinical data, extracting additional radiomic features, adopting a broader range of machine learning algorithms, and conducting multi-dimensional analyses.

Aortic diameter in different planes was closely associated with patients’ prognosis after TEVAR. In the training cohort, patients with an adverse prognosis after TEVAR had a significantly larger aortic diameter than those in the non-adverse-prognosis group (P<0.001). A similar numerical trend was observed in the validation cohort, although the difference was not statistically significant (P=0.105). This result suggests that a larger aortic diameter is associated with a poorer prognosis post-TEVAR. Oishi et al.^15^ found that a maximum distal aortic diameter >40 mm before TEVAR was an independent risk factor for long-term aortic-related death and reintervention in patients with TBAD. Tjaden et al.^16^ reported that a proximal landing zone aortic diameter >40 mm in patients with TBAD was correlated with an increased risk of retrograde Stanford Type-A dissection. A larger aortic diameter is associated with a higher risk of long-term aneurysmal degeneration and subsequent reintervention, warranting more aggressive management strategies.

This study found that elderly patients with TBAD had a higher risk of aortic-related adverse events during the perioperative period and long-term secondary interventional therapy after TEVAR. Currently, there are conflicting findings regarding the impact of advanced age on adverse prognosis after TEVAR. Zhang et al.^17^ performed a meta-analysis using a random-effects model, incorporating 27 studies, and found that the overall reintervention rate after TEVAR in patients with TBAD was 15% (95% CI: 12%~19%), and advanced age was a potential risk factor for this outcome. Elderly patients often have poorer baseline conditions and slower recovery after surgical trauma, leading to an unfavorable prognosis; in contrast, younger patients may present with more severe vascular dissection and poorer visceral perfusion, also resulting in suboptimal clinical outcomes. Optimal preoperative assessment and individualized treatment regimens are the key to achieving favorable outcomes for all patients.

Hypertension is closely linked to the pathogenesis of TBAD, and blood pressure control status affects its prognosis. Zhang et al.^18^ demonstrated that large blood pressure variability was an independent risk factor for aortic-related death in patients with TBAD (OR=4.003, 95%CI: 1.671~9.587), and the rate of false lumen thrombosis at three and six months postoperatively was significantly lower in the high BPV group than in the low BPV group. Diastolic blood pressure may be an influential factor for adverse prognosis after TEVAR. In this study, no statistically significant difference in hypertension was observed between the validation and training cohorts, which may be due to the small sample size. Future research should enroll more participants, conduct multi-center studies, and explore the relationship between hypertension and the prognosis of aortic dissection from multiple dimensions.

In the validation cohort of this study, a lower fibrinogen level was associated with a poorer prognosis after TEVAR, although no such statistical difference was found in the training cohort, possibly owing to the limited sample size. Hypofibrinogenemia may lead to a series of complications, such as bleeding and disseminated intravascular coagulation, resulting in an unfavorable patient prognosis. Previous studies have indicated that hypofibrinogenemia is correlated with in-hospital mortality in patients with aortic dissection. After aortic vascular injury, plasma factor VIIa binds to tissue factor, triggering the activation of the coagulation cascade.^19^

Liu et al.^20^ found that an admission fibrinogen level < 2.17 g/L was associated with an increased risk of in-hospital death (OR=5.527; 95%CI: 1.660-18.401; p=0.005), and hypofibrinogenemia was an independent predictor of in-hospital mortality in patients with aortic dissection. Multivariate analysis^21^ showed that an admission fibrinogen-fibrin degradation product level ≥ 20 mg/ml (OR=7.802, 95%CI: 1.405 to 43.335) was an independent predictor of aortic adverse events, and the authors recommended close monitoring of fibrinogen levels at admission. Paparella et al.^22^ evaluated specific biomarkers of thrombin generation (prothrombin fragment 1.2, F1.2) and plasmin activation (plasmin-antiplasmin complex, PAP) within 32 hours of symptom onset in patients with acute aortic dissection. They found that both F1.2 and PAP were significantly elevated in these patients, indicating robust activation of the coagulation and fibrinolytic systems. Thus, a lower fibrinogen level reflects more severe aortic injury and is associated with a poorer prognosis. The lack of statistical difference in fibrinogen levels in the training cohort of this study may be due to the small sample size, and future studies with a larger sample size are needed to improve the credibility of the findings.

In this study, the D-dimer level was significantly higher in the adverse-prognosis group than in the favorable-prognosis group (P < 0.05). D-dimer reflects the fibrinolytic process during coagulation, and its level is associated with the size of the thrombus and the contact area between the thrombus and blood. Patients with elevated D-dimer levels are prone to postoperative lower extremity deep vein thrombosis and even pulmonary embolism, which can lead to severe perioperative death. Therefore, D-dimer levels are of great significance for prognostic assessment and are correlated with the prognosis of vascular diseases, and they are widely recognized as serum markers reflecting the coagulation and fibrinolytic status of patients.^23^ In a multi-center retrospective cohort study by He et al,^24^ thrombocytopenia (< 140 × 10^9^/L) and a high D-dimer level (> 14.6 µg/mL) were found to increase the in-hospital mortality risk by 3.59-fold in patients with aortic dissection (HR=3.59; 95%CI: 2.00-6.42), leading to the conclusion that a high D-dimer level significantly increases the risk of in-hospital death.

### Strengths of this study:

The strength of this study is that the study is interdisciplinary medical-engineering research combining clinical medicine with engineering technology. Based on abundant clinical imaging and clinical data, the study integrated medical imaging, clinical medicine, and computer science by adopting computer-based image segmentation, feature extraction, and modeling methods, which provide new methods, technologies, and ideas for the research and resolution of challenging problems in the medical field.^25^,^26^ Combining clinical and imaging data, this study developed a nomogram based on non-contrast CT to predict the prognosis of TBAD. By integrating machine learning algorithms, the model can be applied for risk stratification of patients, formulation of individualized treatment and follow-up protocols, early detection of adverse events, and ultimately improvement of postoperative outcomes.^27^

### Limitations:

This was a retrospective study with a relatively small sample size, caused by substantial missing data during follow-up and clinical data collection, which may have selection bias. Also, the small sample size of the validation cohort and the study may compromise the stability and reliability of the model. Only patients with aortic dissection were enrolled in this study, while aortic intramural hematoma and aortic ulcer account for a considerable proportion of clinical cases involving aortic diseases. Moreover, this is a single-center study with only internal grouping; despite randomization and satisfactory results, there is still a risk of selection bias. Additionally, the study reported a 12 months follow-up, but the risk of dissection recurrence may occur more than one year post-operatively. While a one-year follow-up serves as a useful early validation, it may be insufficient to fully assess the predictive value of the model regarding long-term prognosis. Last, the data included in this study has a large time span and may have data heterogeneity, which may limit the model’s generalization ability.

## CONCLUSION

In this study, multiple models were constructed to predict adverse prognosis following TEVAR. The results demonstrated that the combined model integrating clinical and radiomic features achieved optimal performance, accurately identifying adverse prognosis after TEVAR. The model may assist in postoperative risk stratification and may support individualized surveillance and clinical decision-making, although its clinical effectiveness requires prospective external validation.

### Recommendations:

Future high-quality, multi-center, large-sample, prospective clinical studies are needed to identify risk factors with high sensitivity and specificity across different types of adverse events, thereby further guiding clinical diagnosis and treatment with greater precision. Adverse events after TEVAR seriously endanger patients’ lives and health and increase the medical burden. Therefore, exploring risk factors will improve disease assessment and risk stratification, thereby identifying high-risk patients. This will also enable early intervention of adverse events, which is of great significance for optimizing TEVAR treatment strategies and improving long-term prognosis.

### Authors’ contributions:

**JZ:** Literature search, study design and manuscript writing.

**XS, JN, QJ, HD and YS:** data collection, data analysis and interpretation. Critical review.

**JZ:** Manuscript revision and validation and is responsible for the integrity of the study. All authors have read and approved the final manuscript.
